# miRNAs: micro-managers of anticancer combination therapies

**DOI:** 10.1007/s10456-017-9545-x

**Published:** 2017-05-04

**Authors:** Judy R. van Beijnum, Elisa Giovannetti, Dennis Poel, Patrycja Nowak-Sliwinska, Arjan W. Griffioen

**Affiliations:** 1 Angiogenesis Laboratory, Department of Medical Oncology, VUMC - Cancer Center Amsterdam, VU University Medical Center (VUmc), Amsterdam, The Netherlands; 2 Laboratory Medical Oncology, Department of Medical Oncology, VUMC - Cancer Center Amsterdam, VU University Medical Center (VUmc), Amsterdam, The Netherlands; 3 School of Pharmaceutical Sciences, University of Geneva (UNIGE), Geneva, Switzerland

**Keywords:** AngiomRs, Combination therapy, MicroRNA, Tumor angiogenesis, Nanocarriers, Anti-angiogenic therapy, Radiotherapy, Chemotherapy, Photodynamic therapy

## Abstract

Angiogenesis is one of the hallmarks of cancer progression and as such has been considered a target of therapeutic interest. However, single targeted agents have not fully lived up to the initial promise of anti-angiogenic therapy. Therefore, it has been suggested that combining therapies and agents will be the way forward in the oncology field. In recent years, microRNAs (miRNAs) have received considerable attention as drivers of tumor development and progression, either acting as tumor suppressors or as oncogenes (so-called oncomiRs), as well as in the process of tumor angiogenesis (angiomiRs). Not only from a functional, but also from a therapeutic view, miRNAs are attractive tools. Thus far, several mimics and antagonists of miRNAs have entered clinical development. Here, we review the provenance and promise of miRNAs as targets as well as therapeutics to contribute to anti-angiogenesis-based (combination) treatment of cancer.

## Introduction

Cancer progression is dependent on the contribution of different facilitating biological processes, the so-called hallmarks of cancer [1]. One of these hallmarks is the active acquisition of a blood vessel network, by inducing endothelial sprouting of nearby, existing vessels, toward the tumor, a process called angiogenesis. After its initial recognition as potential driver of cancer progression by Judah Folkman, numerous studies have detailed the underlying biology of interacting cells, and pro- and anti-angiogenic growth factors (reviewed by [2–4]). In addition, it has been recognized that the inhibition of tumor angiogenesis may pose an attractive therapeutic option for several reasons. First, the endothelial cells (EC) lining the luminal side of blood vessels are in direct contact with the blood, allowing direct exposure to the administered drugs. Second, it is believed that a limited number of EC support a far larger number of tumor cells, allowing for an ‘avalanche’-effect, following compromise of a small tumor capillary. Third, tumor EC are believed to be genetically more stable than cancer cells, which makes therapeutic effects more predictable. Fourth, only at sites of ongoing tumor angiogenesis EC are activated and phenotypically different from resting EC in the rest of the body, allowing therapeutics to be targeted specifically to these sites of tumor angiogenesis.

Thus far, several anti-angiogenic therapeutics have been approved for the treatment of cancer. The discovery of vascular endothelial growth factor (VEGF) was key to these breakthroughs, but, unfortunately, has also resulted in an only narrow set of available targeted agents. Moreover, while the efficacy of the VEGF targeted agents (antibodies and receptor tyrosine kinase inhibitors; TKIs) proved rather limited, also the initial assumption that resistance to anti-angiogenic therapy was highly unlikely to occur has proven wrong [5–7]. Main culprit to this is the therapeutic focus on only one of many tumor-derived growth factors rather than on selective tumor EC features. Therefore, not only from the point of view of anti-angiogenic therapy, but also from a general cancer management angle, the future therapeutic strategies will rely on personalized combination strategies [5, 8].

As mentioned above, tumor EC are phenotypically different from quiescent EC in other parts of the healthy human body, which is accompanied by a specialized gene expression signature characterized by regulated expression of mRNAs as well as microRNAs (miRNAs) [9–12]. While interference with overexpressed molecules on tumor EC has received considerable attention and has brought some (pre)clinical successes, thus far the therapeutic potential of miRNAs has not been exploited in depth. miRNAs are a class of small, non-coding single-stranded RNA molecules, transcribed from DNA, with the capacity to regulate mRNA levels and activity by binding to mRNA and causing either degradation or translational repression. Their importance to the process of angiogenesis stems mainly from the observations that interference with miRNA biogenesis inhibited angiogenesis [13–15]. Over the last decade, the role of several specific miRNAs in the process of (tumor) angiogenesis has been elucidated (reviewed by [13, 16–20]). Much less explored, however, are the therapeutic opportunities of miRNAs. In this review, we focus on the opportunities of employing miRNAs in (anti-angiogenic) anticancer (combination) therapies.

## miRNAs in tumor angiogenesis

**Fig. 1:**
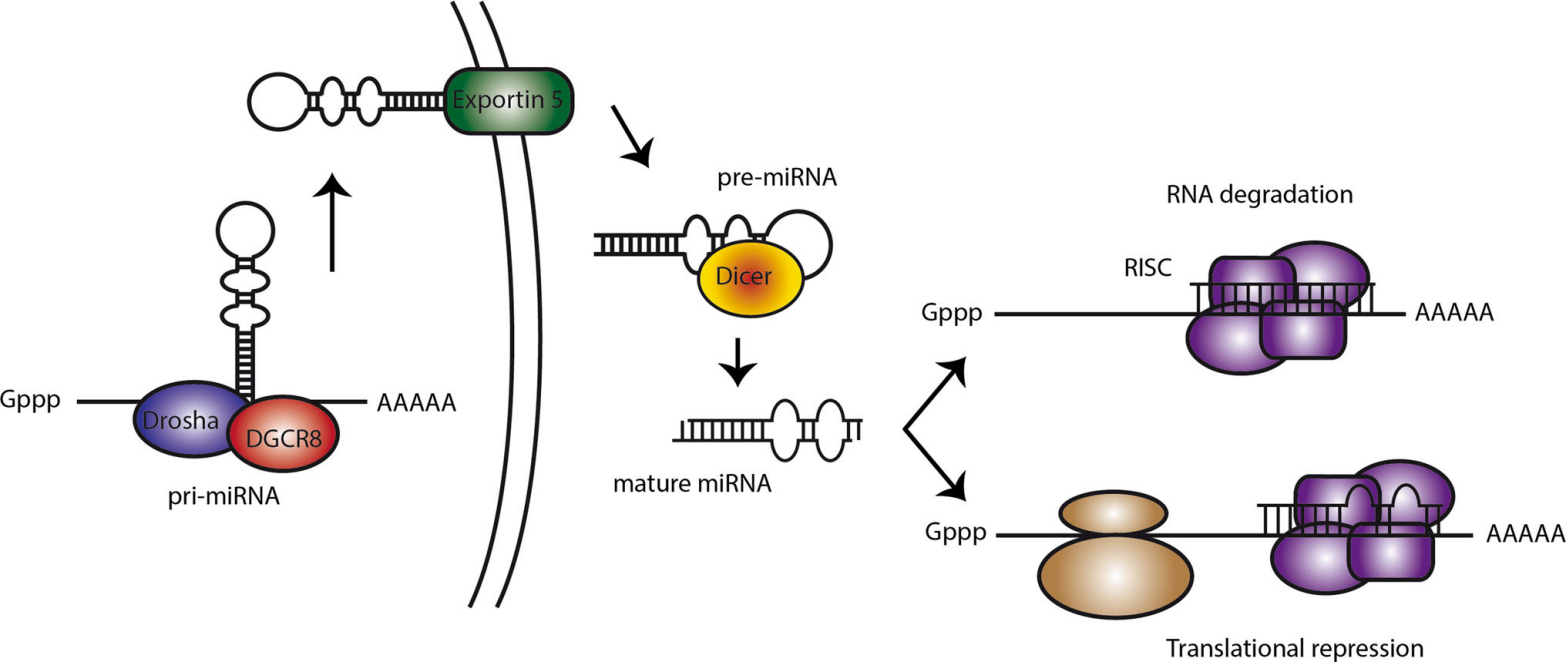
miRNA biogenesis in a nutshell. miRNAs are transcribed from DNA as single-stranded hairpin-looped RNA molecules. The hairpins are cleaved by Drosha and DGCR8, and subsequently exported from the nucleus by Exportin5. In the cytoplasm, Dicer cleaves the stem-loop to generate double-stranded RNA duplexes. One of the strands of the mature miRNA is incorporated in the RNA-induced silencing complex (RISC) where it can complementary pair with the target mRNA, leading to either mRNA degradation in case of full complementarity or translational repression with incomplete base-pairing

MicroRNAs play significant biological roles in modulating gene expression by targeting mRNAs for degradation or translational repression. miRNAs are transcribed from DNA as pri-miRNAs, stem-loop-containing RNA molecules. These are processed in the nucleus by Drosha to single-stem-loop-containing 60–70 nt RNAs. These pre-miRNAs are exported to the cytoplasm and processed by Dicer to generate a mature miRNA duplex. One strand of the mature miRNA incorporates in the RNA-induced silencing complex (RISC) and can subsequently inhibit mRNA translation or target the mRNA for degradation (Fig. 1).

A large number of miRNAs have been implicated in cancer; however, here we briefly discuss several miRNAs explicitly involved in angiogenesis. For in-depth discussions, we refer the reader to dedicated reviews [16–19]. miRNAs are highly expressed in EC, and they can regulate most aspects of vascular development and angiogenesis. The relevance of miRNA biology for angiogenesis was highlighted after knockdown of Dicer, which resulted in the alteration of numerous angiogenesis regulators, including VEGF receptors (VEGFRs), angiopoietin receptors TIE-2/TEK, endothelial nitric oxide synthase (eNOS) and interleukin-8 (IL-8) [15]. A first study performed with a miRNA microarray platform in human umbilical vein endothelial cells (HUVEC) identified 15 highly expressed miRNAs predicted to regulate the expression of receptors for angiogenic factors [12]. More recent miRNA profiling studies identified 200 miRNAs expressed by EC, including 28 miRNAs that were identified in more than 60% of these studies [16]. Among the highly expressed miRNAs in EC, miR-126 promotes angiogenesis in response to VEGF and basic fibroblast growth factor (bFGF) through negative suppression of regulators in signal transduction pathways [16, 21, 22]. In particular, miR-126 exerts pro-angiogenic effects by promoting mitogen-activated protein (MAP) kinase and phosphatidylinositol-3-kinase (PI3K) signaling in response to VEGF and bFGF, through targeting negative regulators of these signaling pathways, including the Sprouty-related EVH-domain-containing protein (Spred-1) and PI3K regulatory subunit 2 [21, 23]. Furthermore miR-126 suppresses metastatic endothelial recruitment, metastatic angiogenesis and metastatic colonization through coordinate targeting of Insulin-like growth factor-binding protein 2 (IGFBP2) and c-Met tyrosine kinase (c-Met) [24]. Other key miRNAs affecting tumor angiogenesis are included in the miR-17–92 cluster, which encodes six mature miRNAs (miR-17, miR-18a, miR-19a, miR-20a, miR-19b, and miR-92a) and represses the levels of the anti-angiogenic molecules thrombospondin 1 (THBS1) and connective tissue growth factor (CTGF) [14]. However, anti-angiogenic actions of members of this cluster have been described as well [19]. miR-17-92 cluster miRNAs target a.o. VEGFR2, tissue inhibitor of metalloproteinase-1 (TIMP-1), eNOS, sphingosine-1-phosphate receptor 1 (S1PR1), cyclin-dependent kinase inhibitor 1A (CDKN1A; p21), Janus kinase-1 (JAK-1) and integrin A5 (ITGA5) [16], and their actions are context- and microenvironment dependent [19]. miR-296 is among the more famous pro-angiogenic miRNA. It targets the hepatocyte growth factor—regulated tyrosine kinase substrate (HGS), which is involved in platelet-derived growth factor receptor (PDGFR), epidermal growth factor receptor (EGFR) and VEGFR signaling. Its expression is induced by angiogenic growth factors and its modulation affects angiogenesis. Inhibition of miR-296 reduced tumor angiogenesis in different models [25–27].

In the class of inhibitory miRNAs, miR-221/222 have drawn major attention. These miRNAs inhibit endothelial migration, proliferation and tube formation via targeting of KIT proto-oncogene receptor tyrosine kinase (c-Kit) expression, and indirectly affecting eNOS [12, 28]. The hypoxia-induced von-Hippel-Lindau (VHL)–hypoxia-inducible factor 1 alpha (HIF1α)–VEGF-axis plays a crucial role in the onset of tumor angiogenesis, and diverse miRNAs have been identified to modulate these players. The related miR-15b and miR-16 as well as miR-124 directly target VEGF expression [29–31], and miR-20b reduces VEGF expression through targeting HIF1α [32]. Additional miRNAs have been identified that directly or indirectly target VEGF [19]. Through negative regulation of VHL, miR-155 increases HIF1α and VEGF expression [33], while miR-21 exerts similar effects through targeting phosphatase and tensin homolog (PTEN), Sprouty 1 (Spry1) and programmed cell death protein 4 (PCDC4) which additionally confers resistance to hypoxia [34, 35].

**Fig. 2:**
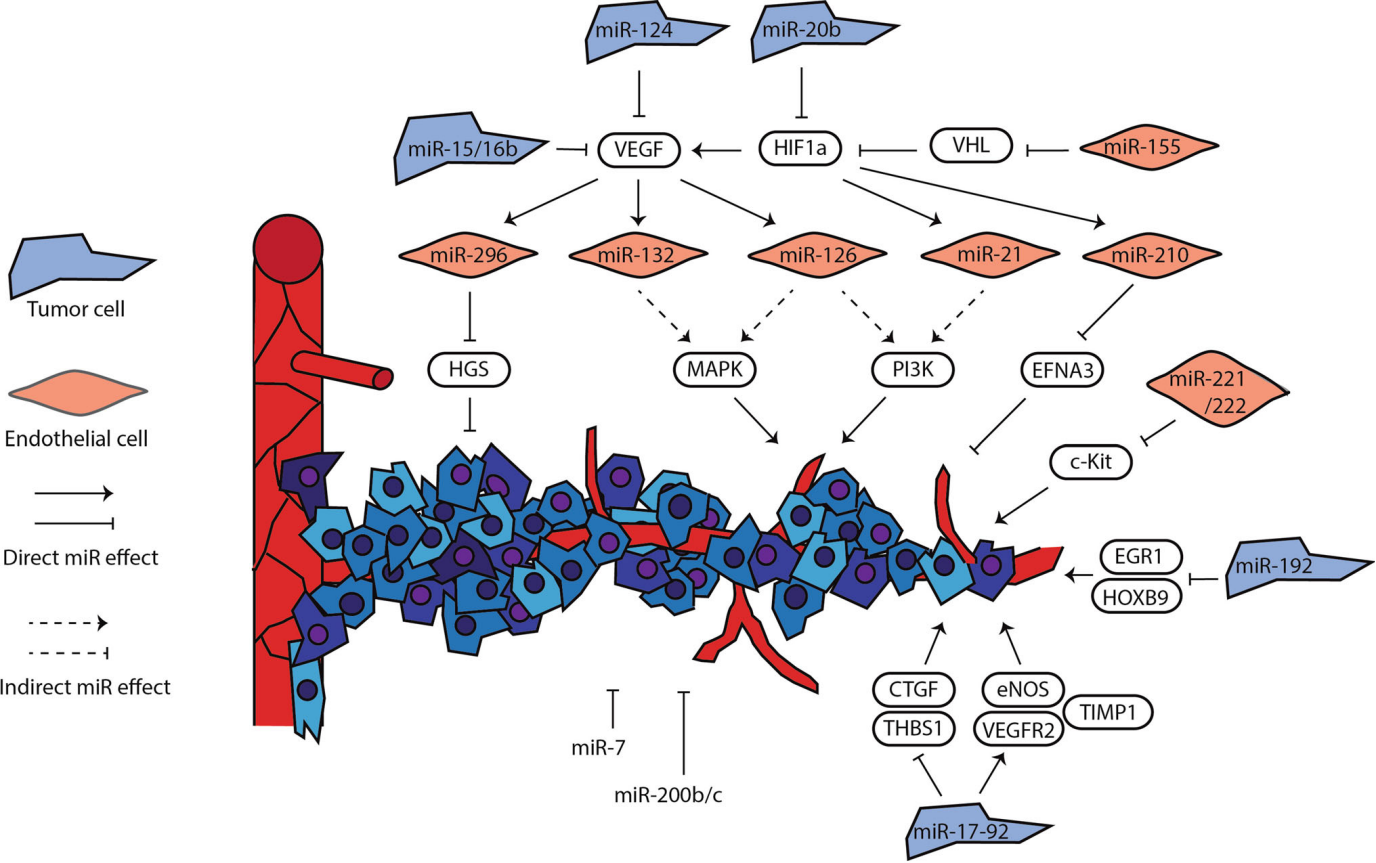
Role of miRNAs in tumor angiogenesis. Schematic impression of miRNAs pivotal to the regulation of tumor angiogenesis, showing their effects on effector proteins. It must be noted that miRNA action is not strictly confined to either tumor cells or endothelial cells

Many studies addressed the pro- or anti-angiogenic functions from a tumor cell perspective, whereas relatively few directly addressed the function of endothelial miRNAs. Using a miRNome-wide lentiviral screen, Babae et al. identified miRNAs involved in endothelial cell proliferation [36]. miR-7 was one of the prime hits and was shown to inhibit EC proliferation, migration and tube formation. Furthermore, it inhibited angiogenesis in vivo in the chorioallantoic membrane of the chicken embryo (CAM) [37] and in two different murine tumor models [36], although molecular effects were not unraveled. Similar effects were demonstrated for miR-200b and miR-200c [38, 39]. In contrast, miR-132 is highly overexpressed in tumor endothelium [40], where it stimulates VEGF driven Ras-Raf-Mek-Erk signaling through targeting RAS p21 protein activator (RASA1; p120 RasGAP), a negative regulator of Ras [40]. miR-132 mimics inhibited tumor angiogenesis in vitro and in vivo [40]. The most important mechanism involved in EC and tumor cell regulation by above-described miRNAs are summarized in Fig. 2.

More recently, several non-repressive roles for miRNAs have been uncovered, e.g., by modulation of translational and transcriptional complexes [41]. Next to that, many other classes of non-coding RNAs have been identified [41] that are believed to play major roles in cancer and tumor angiogenesis, and which may pose attractive therapeutic targets [41–43]. Given the importance of cell-type-specific expression patterns of miRNA, future research will surely provide an even more complex list of endothelial miRNAs that affect other pathways implicated in tumor angiogenesis.

## miRNA-based therapy

### miRNA replacement and miRNA antagonism

From a therapeutic point of view, miRNA and siRNA therapy can offer druggability of otherwise non-druggable targets. By studying cellular pathways and identifying driving factors in an oncogenic process, putative drug targets can be identified [44]. However, should the localization or the spatial availability of the putative target seem limiting, its coding mRNA could be targeted with either siRNA or miRNA.

The major rationale for miRNA replacement therapy is the hypothesis that in cancer a majority of miRNAs is suppressed [45]; thus, inhibition of miRNAs induces tumorigenesis. This, in return, suggests that tumor-suppressive functions of miRNAs prevail over oncogenic functions. miRNA mimics have the identical sequence to the natural endogenous molecule and, as such, will act indistinguishably. Although accumulation of miRNAs in normal cells may be toxic due to overloading of RISC, this has not been studied in detail yet. Furthermore, the impact of enhanced ‘tumor-suppressive’ miRNAs in normal cells is likely limited as there is already a high level of these desired miRNAs present in the cell [46]. In comparison with mRNA replacement therapy, antagonistic miRNA therapy approaches are more cautious and usually target multiple miRNAs simultaneously. However, some argue that it may be safer to inhibit oncomiRs in tumor cells as their expression is already extremely low in normal cells and will therefore hardly affect normal tissues (reviewed by [41, 47–49]).

A proposed advantage of miRNA-based or anti-miRNA-based therapy is that miRNAs tend to target multiple components of related pathways [48–50]. When deregulated in cancer, targeting with (anti-)miRNA therapy can restore cellular balance [49, 51]. Wurdinger et al. refer to such mechanism as a ‘one-hit-multiple-target’ approach [52]. However, a potential threat would be the occurrence of off-target effects as a consequence of sequence similarities.

**Fig. 3:**
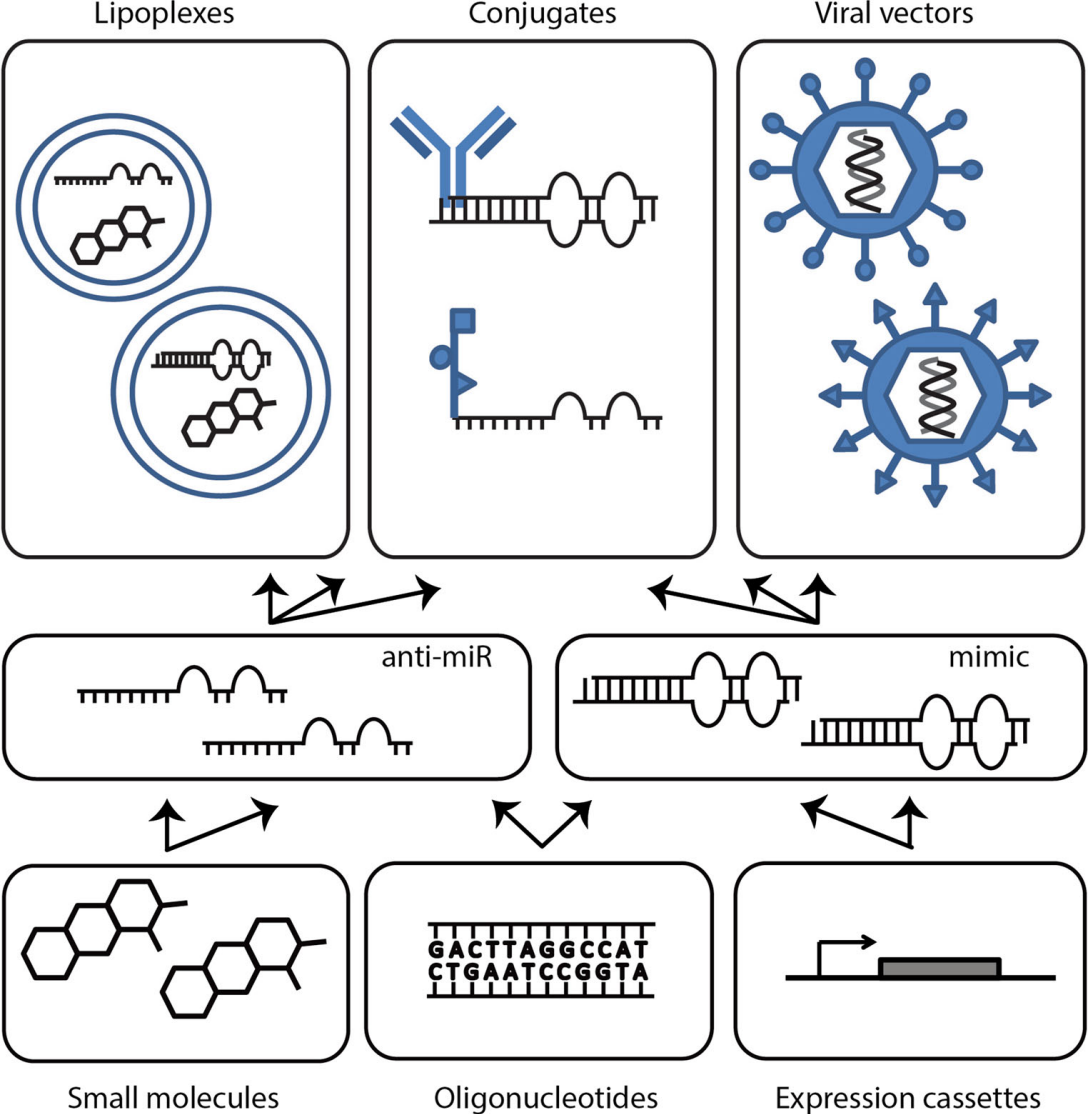
Therapeutic miRNA approaches and delivery methods. Oligonucleotides, expression cassettes and small molecules can fulfill antagonistic or mimicry functions of miRNAs and can be delivered as viral vehicles, lipoplexes or conjugates where desired

In general, miRNA replacement therapy and miRNA reduction therapy (or antisense therapy) can be distinguished. For both, a number of different approaches and moieties have been described, regardless of delivery methods (discussed below; Fig. 3). For both replacement and antisense therapy, the oligonucleotides targeting/mimicking the designated miRNA can be synthesized and introduced into the cytoplasm directly. Depending on the exact delivery system or carrier used, several chemical alterations to stabilize the oligos can be introduced (reviewed by [30, 44, 48, 49]). Second, the mimics or antisense constructs can be embedded in a viral backbone, which allows easier infection of target cells, and/or retargeting of the virus particle through modification of surface anchors [49, 53]. For antisense therapy, such synthetic miRNA sponges have been tested, also expressed from a viral construct [47, 54, 55]. This way, multiple different miRNAs can be scavenged from the cell, either by placing multiple miRNA binding sites in a row, or by incorporating miRNA binding sequences specific for entire miRNA families. A miR-23b sponge construct embedded in a lentiviral vector was tested for reduction of miR-23b levels in different glioblastoma cell lines. By ensuring imperfect base-pairing of endogenous miR-23b to the sponge, the construct was protected from cleavage by Argonaute 2. Functionally, this resulted in reduced angiogenic, invasive and migratory capacities of the glioma cells [54]. Another group explored the adenoviral expression of long non-coding RNAs to scavenge oncomiRs and demonstrated clear anti-tumor effects in both hepatoma and lymphoma models [55, 56]. Finally, a novel class of molecules referred to as small molecule inhibitors of miRNAs (SMIRs) are being exploited. Similarly, small molecule agonists have been identified [53, 57, 58]. Using rational design, small molecules were identified to interact with the secondary structure of miR-96, which resulted in Targaprimir-96 (3) [59]. It is a dimeric small molecule linked with a peptoid backbone, with high affinity to the bulges in the immature forms of miR-96. In a mouse breast tumor model, it showed promising tumor inhibitory activity [59]. Screening of compound libraries identified Rubone [60] as an activator of mir-34a in HCC with pro-apoptotic properties, and Kenpaullone [61] as inducer of miR-182 in cardiac ischemia. Although promising, the development of this class of miRNA targeting agents is still in its infancy, and at present it is not fully elucidated to what extent such molecules are truly sequence-specific, or whether they merely modulate miRNA biogenesis or stability. Furthermore, therapeutic effectivity remains to be proven [47].

It can be stated that miRNA replacement or antagonistic miRNA therapy in itself is a form of combination therapy through its effect on multiple pathways. However, the effects on any pathway it may exert are dependent on different factors, including the activity of that particular pathway.

### miRNA delivery

Key to therapeutic application of miRNAs is the delivery of effective product in the target tissue. Cellular uptake of naked nucleotides is not very efficient [62], although some cells more easily take up miRNAs than others, e.g., glioblastoma multiforme (GBM) [52]. In addition, RNAses in serum or in cells can degrade naked, unmodified miRNA mimics or antagonists. To overcome these issues, two converging approaches have been pursued, namely the chemical modification of the nucleotides themselves and the design of different delivery vehicles. Most well-known modifications of miRNA antagonists are the locked nucleic acids (LNA), 2′-O-methoxyethyl modifications and the phosphorothioate backbones. All such modified oligonucleotides are capable of binding their complementary target; however, there remains a trade-off between increased stability and effective silencing through loading in the RISC complex [47, 49, 62].

Such chemical modifications are less feasible for double-stranded miRNA mimics, as it may induce loss of silencing ability [48]. To this end, different nanoparticles have been formulated to enable in vivo delivery, which can grossly be subdivided in conjugates, lipoplexes (liposomes, polymers) and viral particles [47, 48]. Regarding the non-viral delivery systems, these are usually a combination of lipids to facilitate cell entry and cationic polymers, e.g., polyethyleneimine (PEI) and polyethyleneglycol (PEG). More recently, conjugation to N-acetyl-D-galactosamines (GalNAc) has been explored which uses clathrin-mediated endocytosis for cellular uptake. These conjugates bypass the needs for additional molecules such as lipids [63], and have entered clinical testing for liver diseases [47, 48].

Targeting of miRNA formulations to tumor cells also remains a challenge. Thus far, few targeted nanoparticle formulations have progressed toward the clinic [64], and most systems have relied on trapping in the tumor environment by means of vascular leakage. Nevertheless, more efficient delivery can be expected with targeted nanoparticles, either toward the tumor cells, or toward the tumor endothelium [36, 65]. Furthermore, the loading of both small molecule drugs and miRNA therapeutics in a single nanocarrier offers the potential of efficient delivery and synergistic effects [30].

## miRNA combination therapies

**Fig. 4:**
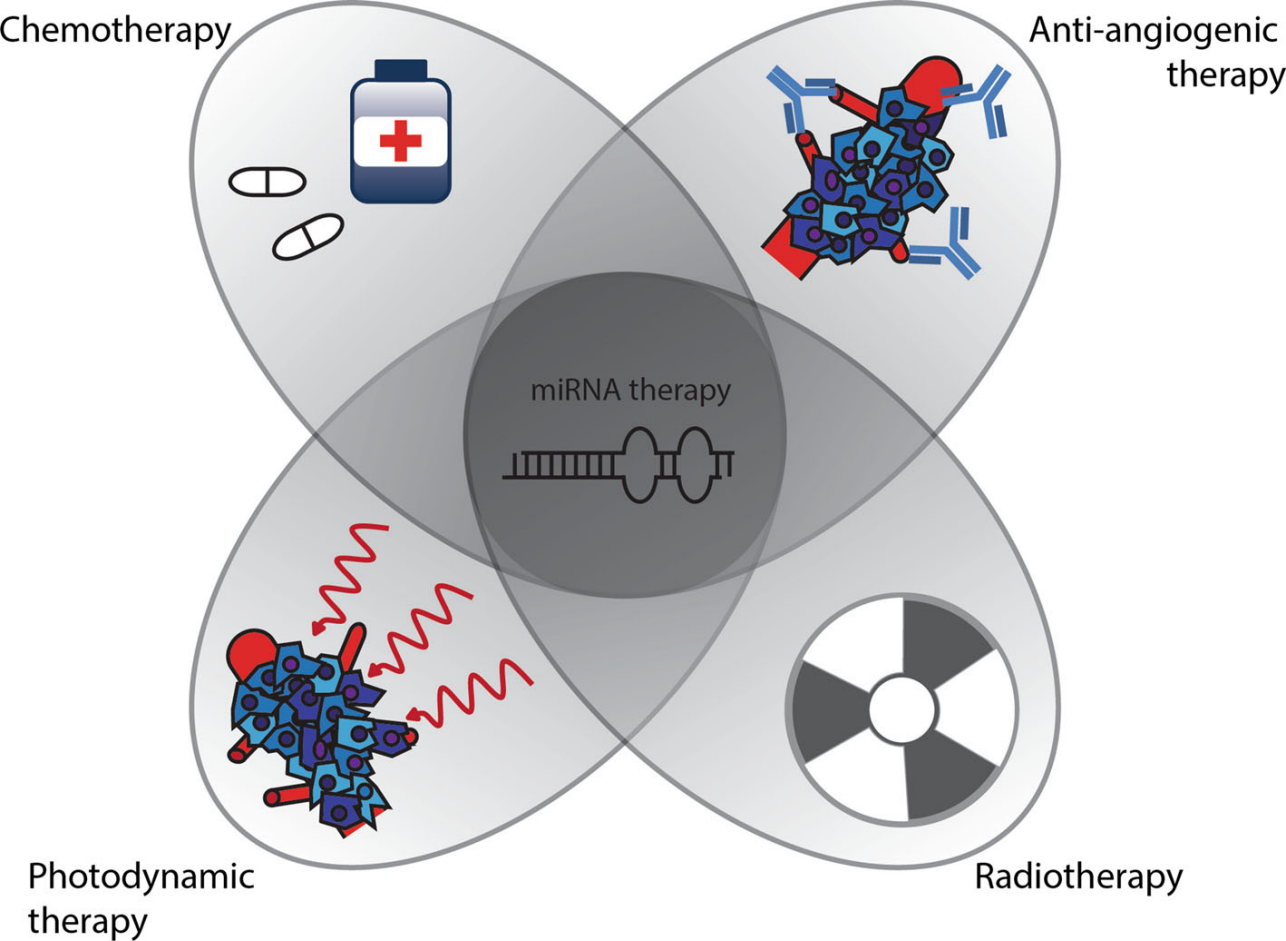
miRNA-based combination therapy. Different conventional therapeutic moieties can be combined with miRNA-based therapies to increase effectiveness. miRNA-based therapy can contribute by increasing sensitivity to conventional therapies, by overcoming resistance and by synergistic activity toward tumor eradication and angiogenesis inhibition

As miRNAs are implicated in most, if not all, signaling pathways in complex disorders, including cancer, they can be an attractive component of combination strategies. These combined regimens are described below and schematically represented in Fig. 4.

## Anti-angiogenic miRNA-based combination therapy

### Combining miRNA-based and anti-angiogenic strategies

Although the multi-valent activity of a single therapeutic miRNA can be considered combination therapy in itself, the combination of miRNAs with alternative therapy is an attractive treatment strategy. However, clinical application of miRNA-based therapy in cancer in general, and with the aim of angiogenesis inhibition in particular, is still in its infancy. Even more so, combining miRNA-based therapy with other therapeutic moieties has only rarely been explored.

A liposomal formulation of miR-34 (MRX34) is currently in clinical trials [66]. miR-34 is a direct target of p53, is suppressed in many different tumor types, and targets different genes involved in malignant progression, such as sirtuin 1 (SIRT1), c-Met, cyclin-dependent kinase 6 (CDK6) and CD44 [67]. In preclinical models, miR-34 replacement synergizes with doxorubicin [68]. Cell proliferation and invasiveness of osteosarcoma cells were reduced, whereas apoptosis was increased. Of note, the sequencing of the two drugs was of importance and showed best effects when both agents were administered simultaneously [68]. Overexpression of miR-34a affects the expression of eNOS and inhibits angiogenesis [67]. In addition, a direct suppression of tumor angiogenesis through inhibition of CD44 expression was demonstrated in a murine model of bladder cancer [69]. Interestingly, the small molecule modulator of miR-34a, Rubone, was identified by screening a natural compound library for affecting a miR-34a luciferase reporter construct [60]. Rubone increased cellular miRNA levels and reduced the expression of miR-34a targets. Rubone exerted anti-angiogenic effects in murine tumor models. Given the sensitizing effect of miR-34a to the VEGFR TKI sorafenib in hepatocellular carcinoma (HCC) [70], the authors suggest combined treatment of HCC with rubone and sorafenib for this disease [60].

A relevant example of combining anti-angiogenic and miRNA-based therapy was provided by Costa et al. [71]. Here, chlorotoxin (CTX)-coupled (targeted) stable nucleic acid lipid particle (SNALP)-formulated anti-miR-21 oligonucleotides were administered in GBM bearing mice. The particles accumulated in the GBM tissue and showed anti-tumor efficacy. Moreover, in combination with the administration of sunitinib, but not temozolomide, this effect was enhanced and accompanied by a reduction of vessel density [71, 72].

In an in vitro study by Passadouro et al., anti-miR-21 was combined with sunitinib and this combination was more effective in inhibiting pancreatic ductal adenocarcinoma (PDAC) cell growth than anti-miR-21 in combination with the standard treatment gemcitabine [73]. A similar example of the power of combining miRNA-based therapy with anti-angiogenic agents was shown in a study by Liu et al. Here, miR-145 mimics were combined with sunitinib on U87 cells which showed additive effects, even though very high concentrations of sunitinib were used [74]. It must be noted, however, that both studies only addressed tumor cell biology and not inasmuch tumor angiogenesis. Although sunitinib is classified as an anti-angiogenic drug, it also directly affects tumor cells [7, 75].

Using a lentiviral miRNA library, miRNAs with anti-angiogenic function were identified, and the most promising miRNA, miR-7, was tested in different tumor models [36]. Using naked miRNA directly injected intratumorally in a subcutaneous Neuro2A model, followed by local electroporation, it was shown that both tumor growth and tumor angiogenesis were inhibited. Its liposomal formulation administered via systemic delivery in a U87 model revealed similar results. However, notable differences in effects were observed as tumor growth inhibition in the Neuro2A model was not accompanied by inhibition of tumor cell proliferation, whereas tumor cell proliferation in addition to vessel density was inhibited in the U87 model [36]. Thus, effects of miR-7 differ between different tumor types. The targeted particles here were aimed at the tumor endothelial marker αVβ3 integrin, thereby effectively classifying the second approach as a combination therapy. As such, the potential for using tumor vasculature targets for (liposomal) miRNA delivery is underscored by this study, as well as by another study from this group combining galectin-1 targeted formulations for VEGFR2 siRNA [65, 76].

The identification of differential miRNAs in highly *versus* poorly angiogenic tumors in large scale patient data sets was reported [77]. miR-192 was noted as a versatile anti-angiogenic miRNA as it can globally downregulate multiple pro-angiogenic pathways, a.o. through regulation of homeobox B9 (HOXB9) and early growth response-1 (EGR1) [77]. Nanoparticle-mediated delivery of miR-192 inhibited tumor growth and tumor angiogenesis in different ovarian- and renal tumor models. This formulation was used in combination with topotecan (which is, however, not seen as a ‘pure’ anti-angiogenic agent) and resulted in additional tumor growth reduction. In addition, the authors noted that the effect of the miR-192 nanoparticles was superior to that of anti-VEGF antibodies [77].

To sum up, although miRNA-based therapeutics in combination with angiostatic therapies have largely been unexplored, ample opportunity exists for future exploration. However, to prevent the occurrence of resistance due to solely targeting the VEGF signaling axis, more insight in the mechanism of action of tumor endothelial miRNAs is warranted.

### Combination of angiostatic miRNA-based therapeutics

An interesting approach of combining miRNA-based therapeutics to inhibit angiogenesis is evident from a study of Lee et al. Runt-related transcription factor 3 (RUNX3) is involved in the destabilization of HIF1α—a major inducer of VEGF production—and thereby prevents tumor angiogenesis [78]. However, RUNX3 is frequently downregulated in tumors. Using different datamining tools, the authors identified miR-30a and miR-145 to be involved in RUNX3 suppression, and therapeutic administration of either miRNA in a Matrigel plug assay in mice inhibited VEGF secretion and vessel invasion. Moreover, the combination showed additive effects in inhibiting angiogenesis [78].

A rather complex approach was presented by Askou et al. who constructed a lentiviral expression cassette containing not only multiple VEGF targeting miRNA clusters, but also a VEGF-antagonistic pigment-epithelium-derived factor (PEDF; SERPINF1) protein construct. These multigenic lentiviral vectors inhibited tube formation of HUVEC in vitro and showed feasibility for targeting mouse retina as a means to target aberrant angiogenesis in CNV [79, 80]. It remains to be proven whether such an approach will hold promise for clinical management of (solid) malignancies.

### miRNAs and anti-angiogenic treatment response

It has been recognized that miRNAs may play an important role in treatment response and therapy resistance. A small number of studies addressed the relationship between miRNA expression and sunitinib response. Sunitinib, a VEGFR TKI, is the first-line treatment for renal cell carcinoma (RCC), with effects on angiogenesis as well as directly on tumor cells. For example, Khella et al. and Garcia-Donas et al. reported on miRNAs able to distinguish between short and long survival in sunitinib-treated RCC, a highly VEGF dependent tumor type [81–83]. miRs-221 and -222 were shown to be upregulated in poor responders, as these miRNAs target the same protein as sunitinib, i.e., VEGFR2, rendering the effect of sunitinib obsolete and making the tumors intrinsically resistant to the treatment. Although this observation would suggest that inhibiting these miRNAs to enhance sunitinib action could provide therapeutic benefit, the opposing actions on tumor cells and EC prevents such an approach. Therefore, these miRNAs might only prove to be of predictive power [81, 82].

In another study, miR-101 was described to be associated with sunitinib resistance in RCC [84]. Furthermore, Merhautova et al. identified a panel of tissue-derived miRNAs that distinguished sunitinib-treated RCC patients based on time to progression, reflecting therapy resistance. miR-484 with as yet undefined biological roles as well as miR-155, an oncomiR with angiogenic properties, were decreased in patients with a better treatment response [85]. A similar association for miR-484 was found in yet another study on predicting sunitinib response in RCC [86], along with miR-628-5p, miR-133a and miR-942. High expression of these miRNAs was associated with poor prognosis. Interestingly, overexpression of miR-942 enhanced the angiogenic mediators matrix metalloproteinase-9 (MMP9) and VEGF, which resulted in enhanced angiogenesis [86]. Finally, it was shown that miR-141 was downregulated in RCC tissue of poor sunitinib responders, which was associated with epithelial-to-mesenchymal transition (EMT) [87]. In vitro, reintroduction of miR-141 reversed EMT.

The use of blood-borne miRNA markers to predict therapy sensitivity to anti-angiogenic agents such as sunitinib has received recent attention. However, different studies identified different (combinations of) miRNAs associated with treatment outcome. A poor response miRNA signature consisted of miR-192, miR-193a-5p and miR-501-3p, whereas a prolonged response model included miR-410, miR-1181 and miR-424* [88]. It must be noted that when combined in these models they hold predictive power, whereas individually these miRNAs only have limited value [88]. Controversially, Shivapurkar et al. report on a more aggressive disease phenotype in colorectal cancer patients who showed decreased circulating levels of the pro-angiogenic miR-296. Low circulating levels of miR-296 were also associated with poor response to capecitabine and sunitinib [89].

Although these studies associate miRNA expression with treatment sensitivity, future studies should reveal whether therapeutic interference with these miRNAs can offer clinical benefit.

## Combination of miRNA therapy and chemotherapy

miRNAs can act as key gene regulators of pro-survival pathways that are used by cancer cells to evade the cytotoxic effects of chemotherapy [90]. In general, miRNA expression levels in tumors can be highly influential for therapeutic response. Therefore, several preclinical studies evaluated the combination of miRNA-based therapeutics with chemotherapy [91]. This paragraph summarizes the most representative examples of the potential role of miRNAs as anti-angiogenesis approach in combination with chemotherapy.

By targeting multiple signaling pathways in the response to hypoxic stress in both EC and tumor cells (Fig. 2), miRNAs can fine-tune angiogenic responses and also modulate the sensitivity to several anticancer agents. The well-studied oncomiR miR-21, which has been identified as one of the most important miRNAs associated with tumor growth and metastasis, is intricately involved in sustaining the mitogen-activated protein kinases ERK 1/2 signaling pathways and thereby enhances HIF1α and VEGF expression [34]. miR-21 expression plays a key role in the resistance to several anticancer agents in preclinical models of different tumor types. In particular, overexpression of miR-21 protected the glioblastoma U87MG cell line against temozolomide-induced apoptosis [92], while the repression of miR-21 expression sensitizes glioblastoma cells to teniposide treatment via inhibition of nuclear factor kappa-B (NF-κB) signaling [93]. Inhibition of miR-21 expression has also been shown to sensitize MCF-7 cells to topotecan by downregulation of the anti-apoptotic protein BCL2 [94]. In addition, inhibition of miR-21 synergistically increased the ability of chemotherapeutic agents and trastuzumab to reduce the viability of erb-b2 receptor tyrosine kinase (ERBB2; HER2)-positive breast cancer cells [95]. Furthermore, low expression of miR-21 has been associated with benefit from gemcitabine treatment in multiple studies in PDAC patients [96–98]. These findings support the ability of miR-21 to interfere with key oncogenic signaling and to shape the tumor microenvironment, including pro-angiogenic processes. miR-21 represents an ideal candidate for a multimodal therapeutic approach, combining miRNA-based gene therapy with anti-angiogenic activity and reversal of chemoresistance toward different anticancer drugs.

Several other angiomiRs have documented influence on chemotherapy sensitivity. A recent study in bone marrow samples from 114 children with acute lymphoblastic leukemia (ALL) showed that miR-210 expression was significantly lower in patients suffering from relapse and therapy failure than in other patients [99]. Moreover, increasing miR-210 expression enhanced the response of ALL cells to daunorubicin-dexamethasone-L-asparaginase and daunorubicin-dexamethasone-L-vincristine combinations. Similar results were observed in glioblastoma cells, where forced overexpression of miR-210 led to a reduction in resistance to temozolomide [100]. These data support a role for miR-210 as a prognostic factor and predictor of drug sensitivity. In addition, it highlights a potential for therapeutic interference in miR-210 to accomplish chemosensitization.

The regulation of the VEGF/PI3K signaling pathway by miR-126 has been associated with increased sensitivity to adriamycin and vincristine in lung cancer cells. These results suggest that future therapeutic approaches increasing endogenous miR-126 levels should be compared with monoclonal antibody-based anti-VEGF therapies, such as bevacizumab, since they might not only regulate the integrity of the normal vascular system [101], but also sensitize cancer cells to chemotherapeutics [102]. miR-126 is located in an intron of the EGFL7 (EGF-like domain multiple 7) gene, which is frequently silenced by methylation. As such, it suppression can be reversed by the application epigenetic therapeutics such as DNA methyltransferase and histone deacetylase (HDAC) inhibitors [103, 104], thereby increasing sensitivity to chemotherapy. In another study it was shown that entinostat, an HDAC inhibitor, increases the expression of miR-203 and miR-542-3p, which target surivin expression, responsible for paclitaxel resistance [105]. Additional tumor-suppressive miRNAs silenced by epigenetic events have recently been identified [106], providing opportunities in combining epigenetic therapeutics for miRNA regulation in combination with other agents [104].

Not all miRNA involvement is unambiguously linked to a certain therapeutic effect. The benefits of an anti-angiogenic outcome from targeting the miR-17-92 cluster might be neutralized by the negative effects of the same miRNAs on the activity of cytotoxic drugs [107]. In particular, expression of miR-17-92 has been negatively correlated with cisplatin resistance in non-small cell lung cancer (NSCLC). As a result of low expression of miR-17 and miR-92, NSCLC cells express high levels of transforming growth factor beta receptor-2 (TGFβR2), CDKN1A and RAD21, which contribute to cisplatin resistance through modulation of the repair of DNA damage [107]. In colorectal cancer cells, however, 5-fluorouracil (5-FU) dose-dependently decreased the expression of miR-17-92 resulting in increased the expression of the angiogenesis inhibitor THBS1 mRNA [108].

As described above, miR-221 and miR-222 target c-Kit and thereby act as negative regulators of angiogenesis [12]. However, miR-221 can also specifically promote cancer cell proliferation through modulation of the mTOR (mammalian target of rapamycin) pathway [109]. As such, synthetic inhibitors of miR-221 have been explored in combination with conventional cytotoxic drugs. Synergistic induction of apoptosis by several classical anticancer drugs such as cisplatin, temozolomide and gemcitabine in preclinical models of glioma, breast and pancreatic cancer was demonstrated [110–112]. Studies in breast and oral squamous cell carcinoma cells showed that silencing of miR-221 enhanced the sensitivity to tamoxifen and adriamycin through upregulation of the tissue inhibitor of metalloproteinase-3 (TIMP3) [113, 114]. TIMP3 is a potent inhibitor of angiogenesis via a direct interaction with VEGFR2 and inhibition of proliferation, migration and tube formation, as well as apoptosis induction in EC [115]. Therefore, the combination of anti-miR-221 and cytotoxic drugs seems a promising approach to improve the outcome of chemotherapy through modulation of angiogenesis, although the contradictory roles of miR-221/222 in EC and tumor cells warrant caution.

Collectively, these data provide a rationale for using therapies targeted against specific miRNAs in order to improve the effects of many chemotherapeutic drugs, and in particular to potentiate their anticancer activity through modulation of angiogenesis. However, sometimes contradicting effects in different tumor compartments and cell types precludes exploitation for therapeutic interference. Moreover, miRNAs can have opposite effects toward the same anticancer agent in different tumor types, as clearly demonstrated by the miRNA profiling of the NCI-60 panel, a panel of 60 diverse human cancer cell lines [91]. Similarly, several miRNAs can have both pro- and anti-angiogenic properties and can alter the potency of various anticancer agents differently in the same cancer cell [19]. These controversial results suggest that the relationship between the function of miRNAs and chemotherapy sensitivity/resistance is highly complex, and should prompt further studies on how cellular miRNA expression affects the response of a cancer to cytotoxic therapies as well as the modulation of tumor microenvironment, focusing on angiogenesis.

## Combination of miRNA therapy and radiotherapy

The clinical outcome of radiotherapy significantly improves when combined with chemotherapy or targeted therapy [116–118]. Approximately 50% of all patients with cancer will receive radiotherapy either alone or in combination with other therapies. Unfortunately, not all patients will respond to this type of treatment. Hypoxia [119], abnormal DNA damage response [120] and intrinsic resistance [121] are the main biological explanations for this phenomenon. Recently, the combination of radiotherapy with anti-angiogenic drugs has revealed benefit in preclinical and clinical studies [117, 122, 123]. Anti-angiogenic drugs possibly improve radiotherapy as a result of tumor oxygenation due to vascular normalization, and inhibition of radiotherapy induced angiogenic growth factors [122, 124–126].

Despite the observation that the combination of radiotherapy with anti-angiogenesis has yielded promising results, still many patients develop local recurrence or distant metastasis. Consequently, these patients experience severe unnecessary side effects from the generally more toxic combinations. In addition, rapid resistance to current targeted anti-angiogenic drugs occurs. For this reason, alternative strategies to target angiogenesis in combination with radiotherapy are necessary.

miRNAs can inhibit or stimulate tumor vessel formation as detailed above. Abnormal vascular structures in solid tumors result in hypoxia. Generally, hypoxia is tumor specific [127]. Cells of many different tumor types under hypoxic conditions have specific miRNA profiles [19, 29, 128, 129]. Furthermore, miRNAs induced under hypoxic conditions can stimulate angiogenesis [13]. Successful clinical trials and the development of better delivery systems have created opportunities for miRNAs as potential radiosensitizers. As such, we here focus on the potential radiosensitizing effect of miRNAs in relation to tumor vessel formation and hypoxia.

miR-210 appears to be a key miRNA player in hypoxia in solid tumors. miR-210 both promotes hypoxia and is induced under hypoxic conditions [130–132]. By targeting negative regulators of VEGF and NOTCH1 signaling such as Ephrin A3 (EFNA3) and phosphotyrosine phosphatase 1B (PTP1B), its overexpression promotes the angiogenic phenotype [129, 131, 133]. As effective radiotherapy relies on the presence of sufficient oxygen, hypoxic conditions as well as a tortuous vasculature due to excessive angiogenesis can reduce a radiotherapy response. Consequently, inhibition of miR-210 in hypoxic tumors could restore abnormal tumor blood vessels, improve tumor oxygenation and thus sensitize tumors to radiotherapy. This radiosensitizing effect of miR-210 inhibition has indeed been observed in different tumor types in vitro and in vivo, and CD31 staining of tissues treated with combined miR-210 inhibition and radiotherapy revealed less blood vessels compared to their controls [131, 132, 134, 135]. In general, miR-210 expression promotes angiogenesis in response to hypoxia, indicating a promising therapeutic potential for miR-210 inhibition in combination with radiotherapy. However, these results were only observed under hypoxic conditions, and as such can explain the paradoxical positive association between miR-210 expression levels and chemosensitivity in hematological malignancies as described in the previous section.

Another hypoxia-induced miRNA is miR-155, which has also been shown to play a role in angiogenesis [33, 136]. By targeting the forkhead transcriptional factor family member FOXO3a, a well-known regulator of cell survival in vivo [33], miR-155 enhances neovascularization. In addition, miR-155 exerts angiogenic control through the transcription factor ELK3 [137] and E2F2 [136]. Like for miR-210, knockdown of miR-155 can sensitize tumors to radiotherapy by inhibition of the angiogenic response to hypoxia. This was shown in NSCLC [138], nasopharyngeal carcinoma [139], although the opposite was true for breast cancer [140]. Together these findings indicate that a possible anti-angiogenic effect of miR-155 inhibition can sensitize hypoxic tumors to radiotherapy, although effects are likely heavily dependent on tumor type and microenvironmental factors.

The activating effects of AKT and PTEN by inhibition of miR-221/222 have demonstrated promising radiosensitizing effects in glioma and colorectal cancer cells. Although much is unknown about miR-221/222 in relation to radiotherapy sensitivity, the many anti-angiogenic targets of miR-221/222 and its synergizing effect raise a potential therapeutic approach for combination with radiotherapy. Moreover, given the favorable effects of miR-221/222 inhibition with chemotherapy, targeting this angiomiR could prove beneficial in a wide array of combination therapies.

miRNA replacement as radiosensitizing therapeutic approach has been successfully studied for different miRNAs in vitro and in vivo [141–143]. Generally, miRNAs selected for these studies are related to the inhibition of abnormal DNA damage response, a well-established resistance mechanism for radiotherapy [120]. miR-34a, currently tested in the clinic as MIRX34, has shown radiosensitizing effects in NSCLC by direct binding to the 3′ untranslated region of DNA damage response gene RAD51 in vivo [142]. Although the synergizing effect of miR-34a was determined by its effect on DNA damage response, miR-34a, as mentioned above, has many different targets involved in angiogenesis. As such, its radiosensitizing effect could also be linked to inhibition of angiogenesis.

As presented here, many studies reveal promising results using miRNA replacement or miRNA antisense therapy as radiosensitizers. Previous studies revealed that the effect of anti-angiogenic drugs in combination with radiotherapy highly depends on the dose and treatment schedule. As such, future studies should reveal optimal combinations of radiotherapy with miRNA therapeutics.

## Combination of miRNA therapy and photodynamic therapy

Photodynamic therapy (PDT) is a form of treatment based on the systemic administration of a photosensitive agent, called a photosensitizer, and its local activation by exposure to wavelength-specific light [144]. Selectively exciting the photosensitizer with an appropriate light wavelength and sufficient intensity results in reactivity with environmental oxygen to produce highly reactive oxygen species (ROS) that damage surrounding tissues and lead to vaso-occlusion [145]. PDT is clinically used in the treatment of various superficial cancer types, however, with limited success [146, 147]. The major limitation of PDT is the secondary induction of angiogenic pathways in response to tissue hypoxia and tissue damage, resulting from blood vessel closure, consequently leading to enhanced tumor growth after treatment [148, 149]. Therefore, a promising strategy to overcome these secondary effects is through the combination of PDT with anti-angiogenic drugs [150–152].

PDT-induced cytotoxicity and resistance mechanisms have been broadly investigated [144, 153], while very few miRNA expression patterns specific to PDT are available. The tailored combination of PDT with anti-angiogenic miRNA candidates seems to be an attractive option for more effective cancer treatment. Bach et al. conducted miRNome analysis following PDT with polyvinylpyrrolidone hypericin (PVPH) as a photosensitizer [154]. The authors observed the increase in the expression of several miRNAs (i.e., miRNA-1260b or miRNA-1280). This expression was dependent on the cell type and the incubation time with the photosensitizer, but the role of the specific miRNAs in the PDT-driven cytotoxicity still needs to be explained. Another study on PDT in HeLa cells, using talaporfin sodium as a photosensitizer, revealed significantly increased expression levels of miR-210 and miR-296 [155]. These miRNAs were found to play a role in apoptosis induction, depicting a possible post-PDT mechanism. Moreover, in analogy to PDT-induced angiogenesis [156], these miRNAs are also known for their pro-angiogenic effects. Interestingly, in an in vivo human glioblastoma model, photofrin-based PDT followed by miR-99a transfection significantly increased the induction of apoptosis [157]. Mechanistically, this was suggested to be a consequence of downregulation of fibroblast growth factor-3 (FGFR3) and PI3K/AKT signaling pathways, known to be linked with induction of cell proliferation inhibition and molecular mechanisms of apoptosis.

The above-mentioned studies suggest that combination of PDT with miRNA treatment can have clinical application for the treatment of cancer. It is therefore important to perform systematic searches for miRNAs that are regulated by defined vaso-occlusive PDT conditions. The consequent cellular responses could potentially identify the miRNA signaling based targets that can be further addressed for clinical application.

## Conclusions and outlook

The complete picture of miRNA regulation in cancer and tumor angiogenesis is highly complex. Moreover, depending on the context and tumor type, specific miRNAs can have opposing effects. Several miRNAs show both pro- and anti-angiogenic properties and can alter the potency of various anticancer agents differently in the same cancer cell. Nevertheless, miRNAs and other non-coding RNAs offer a vast landscape of targeting opportunities. In this review, we tried to sketch such a landscape, although this of course remains an artist impression for we cannot be exhaustive. We have highlighted the most important miRNA players in angiogenesis and their relevance in the application of conventional anticancer therapy. We expect that in the upcoming field of immunotherapy [158, 159] the importance and relevance of non-coding RNAs will also present itself.

The path from functional miRNA discovery toward clinical application entails a number of steps to be implemented, including (1) preclinical target identification and unraveling of effector mechanisms and pathways, (2) chemical modification and delivery systems, (3) disease targeting, (4) immunogenic and/or toxic effects, (5) dose optimization and therapeutic effects and (6) rational optimization of the combination strategies [150, 160, 161].

Little is currently known on the risk of resistance to miRNA-based therapeutics, although it is not unreasonable to believe that it can develop. Two major mechanisms are expected to be responsible for such a scenario. First, the cellular uptake and delivery may become hampered through endosomal escape, enhanced enzymatic degradation, increased efflux and/or downregulation of the surface molecules facilitating targeting. Second, mechanistic action of the miRNA therapy may be counteracted by cellular up/downregulation of targeted miRNAs or mRNAs. So far, little evidence has been presented for mutations in targeted miRNA genes.

Taken together miRNAs play important role in pathological (tumor) neovascularization by multiple triggers. The field of miRNA-combinations is still evolving, and continuous discovery of novel endothelium-specific targets together with promising methods for drug combination optimizations and nanocarrier systems will guide to successful clinical combinatorial approaches. Although several challenges remain, there is ample rationale for using therapies targeted against specific miRNAs in order to improve the effects of many conventional drugs and treatments, and in particular to potentiate their anticancer activity through modulation of angiogenesis.

## Abbreviations

AKT: AKT serine/threonine kinase
ALL: Acute lymphoblastic leukemia
CDK: Cyclin-dependent kinase
CDKN1A: Cyclin-dependent kinase inhibitor 1A
c-Kit: KIT proto-oncogene receptor tyrosine kinase
c-Met: MET proto-oncogene receptor tyrosine kinase
EC: Endothelial cell(s)
EMT: Epithelial-to-mesenchymal transition
eNOS: Endothelial nitric oxide synthase
FGF: Fibroblast growth factor
GBM: Glioblastoma multiforme
HCC: Hepatocellular carcinoma
HIF1α: Hypoxia-inducible factor -1 alpha
HUVEC: Human umbilical vein endothelial cells
IL: Interleukin
LNA: Locked nucleic acid
MAP(K): Mitogen-activated protein(kinase)
miRNAs: MicroRNAs
MMP: Matrix metalloproteinase
NF-κB: Nuclear factor kappa-B
NSCLC: Non-small cell lung cancer
PDT: Photodynamic therapy
PI3 K: Phosphatidylinositol-3-kinase
PTEN: Phosphatase and tensin homolog
RCC: Renal cell carcinoma
RISC: RNA-induced silencing complex
RUNX3: Runt-related transcription factor 3
THBS1: Thrombospondin 1
TIMP: Tissue inhibitor of metalloproteinase
TKIs: Receptor tyrosine kinase inhibitors
VEGF: Vascular endothelial growth factor
VEGFR: VEGF receptor
