# Erratum to: miRNAs: micro-managers of anticancer combination therapies

**DOI:** 10.1007/s10456-017-9564-7

**Published:** 2017-07-19

**Authors:** Judy R. van Beijnum, Elisa Giovannetti, Dennis Poel, Patrycja Nowak-Sliwinska, Arjan W. Griffioen

**Affiliations:** 1 Angiogenesis Laboratory, Department of Medical Oncology, VUMC - Cancer Center Amsterdam, VU University Medical Center (VUmc), Amsterdam, The Netherlands; 2 Laboratory Medical Oncology, Department of Medical Oncology, VUMC - Cancer Center Amsterdam, VU University Medical Center (VUmc), Amsterdam, The Netherlands; 3 School of Pharmaceutical Sciences, University of Geneva (UNIGE), Geneva, Switzerland

## Erratum to: Angiogenesis (2017) 20:269–285 DOI 10.1007/s10456-017-9545-x

In the original publication, the article was published with the copyright Springer Science+Business Media. However, it has to be published with free open access agreement. It has also been updated in the original article. 

